# DICOM segmentation and STL creation for 3D printing: a process and software package comparison for osseous anatomy

**DOI:** 10.1186/s41205-020-00069-2

**Published:** 2020-07-31

**Authors:** Takashi Kamio, Madoka Suzuki, Rieko Asaumi, Taisuke Kawai

**Affiliations:** Department of Oral and Maxillofacial Radiology, The Nippon Dental University, 1-9-20 Fujimi-cho, Chiyoda-ku, Tokyo, 102-8159 Japan

**Keywords:** 3D printing, Computed-aided design, DICOM image, FDM 3D printer, Oral and maxillofacial surgery, Patient-specific, STL file

## Abstract

**Background:**

Extracting and three-dimensional (3D) printing an organ in a region of interest in DICOM images typically calls for segmentation as a first step in support of 3D printing. The DICOM images are not exported to STL data immediately, but segmentation masks are exported to STL models. After primary and secondary processing, including noise removal and hole correction, the STL data can be 3D printed. The quality of the 3D model is directly related to the quality of the STL data. This study focuses and reports on the DICOM to STL segmentation performance for nine software packages.

**Methods:**

Multidetector row CT scanning was performed on a dry human mandible with two 10-mm-diameter bearing balls as a phantom. The DICOM image file was then segmented and exported to an STL file using nine different commercial/open-source software packages. Once the STL models were created, the data (file) properties and the size and volume of each file were measured, and differences across the software packages were noted. Additionally, to evaluate differences between the shapes of the STL models by software package, each pair of STL models was superimposed, with the observed differences between their shapes characterized as the shape error.

Results: The data (file) size of the STL file and the number of triangles that constitute each STL model were different across all software packages, but no statistically significant differences were found across software packages. The created ball STL model expanded in the X-, Y-, and Z-axis directions, with the length in the Z-axis direction (body axis direction) being slightly longer than that in the other directions. The mean shape error between software packages of the mandibular STL model was 0.11 mm, but there was no statistically significant difference between them.

**Conclusions:**

Our results revealed that there are some differences between the software packages that perform the segmentation and STL creation of the DICOM image data. In particular, the features of each software package appeared in the fine and thin areas of the osseous structures. When using these software packages, it is necessary to understand the characteristics of each.

## Background

Digital Imaging and Communications in Medicine (DICOM) is the leading standard around the world within the medical imaging information field. Three-dimensional (3D) printing from DICOM images has become easier with the advancement of technologies such as medical engineering, imaging engineering, and the evolution and decreasing costs of hardware and software. Patient-specific 3D models are now being used in many situations within the oral and maxillofacial surgery fields, including education, surgical planning, and surgical simulation [1–4].

3D printing of DICOM images works with stacked 2D images that must be segmented to a data format required by the 3D printer. For this purpose, DICOM images are now being segmented to a 3D computer-aided design (CAD) format for intermediate data, on which primary processing, such as region of interest (ROI) setting, can be performed. Of the approximately 100 file formats of 3D CAD data that are used as 3D native files and intermediate files [5], a stereolithography (STL) file format is the most commonly used format for 3D printing [6, 7]. There are many commercial (fee-based) and open-source (free-of-charge) software packages for segmenting DICOM images to STL data, all of which can run on a general-purpose personal computer (PC).

Our 3D printing system uses a fused deposition modeling (FDM) desktop 3D printer, which is suitable for fabricating solid 3D models. We utilize 3D models in oral and maxillofacial surgery that operate on osseous structures, such as tooth extraction, jaw cysts, jaw bone tumors, and jaw deformities [8]. As described in a previous report [9], even in the oral and maxillofacial fields, surgeons use their anatomical knowledge and experiences to understand the anatomical structures on preoperative images or on the patient intraoperatively. 3D models are particularly useful because curved surfaces and minute areas are difficult to understand via a PC display. Compared to the number of case reports utilizing 3D models, there have been very few reports on 3D printing know-how, that is, creating “necessary and sufficient” 3D printable data. We therefore needed to learn 3D printing through trial and error. In 2018, we reported in 3D Printing in Medicine a “one-stop 3D printing lab” that enables data creation for 3D printing in one facility [8]. In this lab, it is possible to fabricate “inexpensive” 3D models, where the first step toward 3D printing is segmenting the DICOM images and creating the STL (3D CAD) model. We have found that the shape of the created STL model varies slightly from one software package to another. The quality of the STL data affects the 3D printing, and improper STL data can lead to the unsuccessful fabrication of 3D models [10].

In this study, we focus on the performance of software packages that segment and create DICOM images to STL data and report on a comparative analysis across the packages to understand the differences of each and their characteristics. The purpose of this study was to investigate the points to be noted in creating STL data for 3D printing to promote the use of 3D models in the field of oral and maxillofacial surgery.

## Methods

In this study, PC applications that export DICOM images into STL file format data (or that offer a segmentation function) are referred to as “STL data” “software packages”, and a 3D surface model (virtual 3D model) created from STL data is referred to as “an STL model”.

**Fig. 1:**
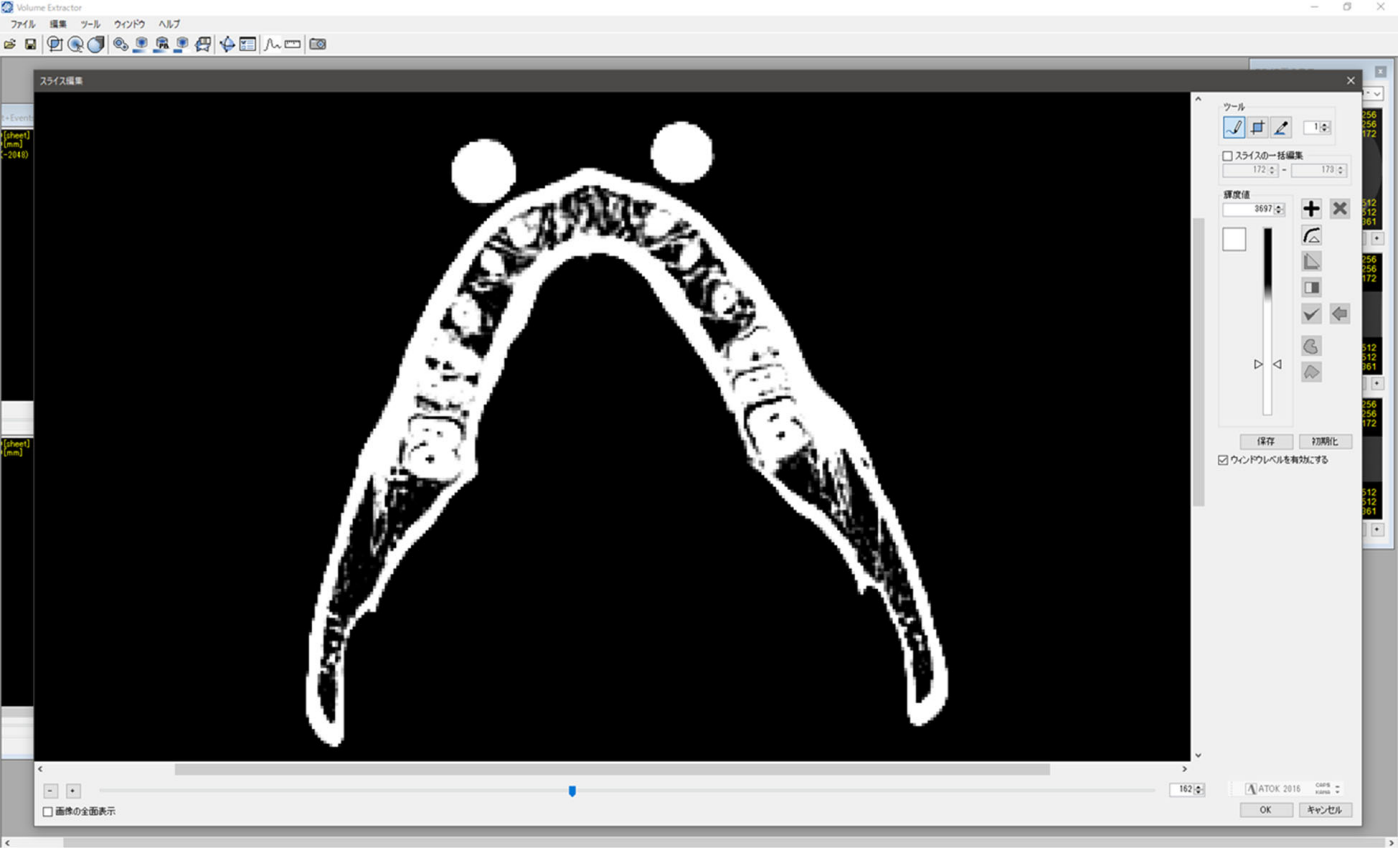
Axial view of the dry human mandible with two 10-mm-diameter aluminum bearing balls attached to the left and right mental regions as phantoms displayed on VE3. The CT value was measured by IMJ, and the mean value inside each ball was approximately 350 HU

Multidetector row CT (MDCT) scanning was performed on a dry human mandible with two 10-mm-diameter aluminum bearing balls attached to the left and right mental regions as phantoms (Fig. 1). A gap of approximately 1 mm was maintained between the mandible and ball to aid segmentation with a PC. The DICOM images were exported to STL files in binary format using one of these packages. First, the data (file) size and volume of the STL file that constitutes each STL model were evaluated. Next, all mandible STL models were compared to assess whether there were differences in the shapes of the created STL models that could be correlated with differences in software and, if so, which areas were affected. In addition, the result of a morphological change by reducing the data size of the mandible STL model is discussed.

### MDCT scanner and scanning parameters

The phantom was scanned with a 64-slice MDCT (Aquilion 64, Canon Medical Systems Corp. formerly Toshiba Medical Systems, Tochigi, Japan) with the following scanning parameters: 120 kV tube voltage, 50 mAs, 0.5 mm slice thickness, 240 mm FOV, 512 × 512 matrix, and convolution kernel FC30.

As a reconstruction filter for MDCT, FC30, a high-resolution reconstruction image filter used for bone imaging in clinical practice, was used [11].

### Software used for segmenting DICOM images to STL data and the evaluation procedure

#### DICOM to STL data segmentation

Table 1 shows details of the nine software packages available for this purpose that can be run on a PC. ROIs and thresholds were set for each software package to create the STL model. The threshold for binarization was set to 350 as a voxel value (brightness value) corresponding to a CT value across all software packages. For packages that support a parameter for resolution, it was set to the “maximum”. Some software packages were able to reduce the data size when segmenting to STL data; for these packages, “no data size reduction (or minimum)” or “no smoothing” was selected. The software simply sets the threshold for binarization and does not add any other functions such as brush/touch-up.

**Table 1:** STL data segmentation software packages used in this study (Accessed 15th Dec 2019)

Software package (Abbreviations)	Version	Developer/Provider
		Web site
**3D Slicer** (3DS)	4.10.2	Surgical Planning Lab, Harvard Medical School, Harvard University, MA, USA
		http://www.slicer.org
**3DView** (3DV)	1.2	RMR Systems Ltd., East Anglia, UK
		http://www.rmrsystems.co.uk/volume_rendering.htm
**Image J** (IMJ)	1.48	National Institutes of Health, Bethesda, MD, USA
		https://imagej.nih.gov/ij
**InVesalius 3** (IN3)	3.1.1	Renato Archer Information Technology Centre, São Paulo, Brazil
		https://invesalius.github.io
**Mimics**^a^ (MCS)	22.0.0.524	Materialise, Leuven, Belgium
		https://www.materialise.com/en/medical/mimics-innovation-suite/mimics
**The Medical Imaging Interaction Toolkit** (MIT)	2018.04.2	German Cancer Research Center, Heidelberg, Germany
		http://mitk.org
**OsiriX Lite** (OSX)	11.0.0	Pixmeo SARL, Geneva, Switzerland
		http://www.osirix-viewer.com
**Seg3D** (S3D)	2.4.4	Scientific Computing and Imaging Institute, Salt Lake City, Utah, USA
		http://www.sci.utah.edu/cibc-software/seg3d.html
**Volume Extractor 3.0**^a^ (VE3)	3.6.0.7	i-Plants Systems, Iwate, Japan
		http://www.i-plants.jp/hp/products/ve3

^a^Commercial software

STL data can store information in two different ways, namely, binary encoding and ASCII encoding. The two formats contain the same information regarding the model, but the binary format is much more compact; it will produce smaller files (but they should work the same). In this study, the STL data were exported in a binary format. ImageJ, by default, does not have an STL segment function, so a plugin tool (3D Viewer, https://imagej.nih.gov/ij/plugins/3d-viewer) was installed.

#### 3D coordinate system and measurement

**Fig. 2:**
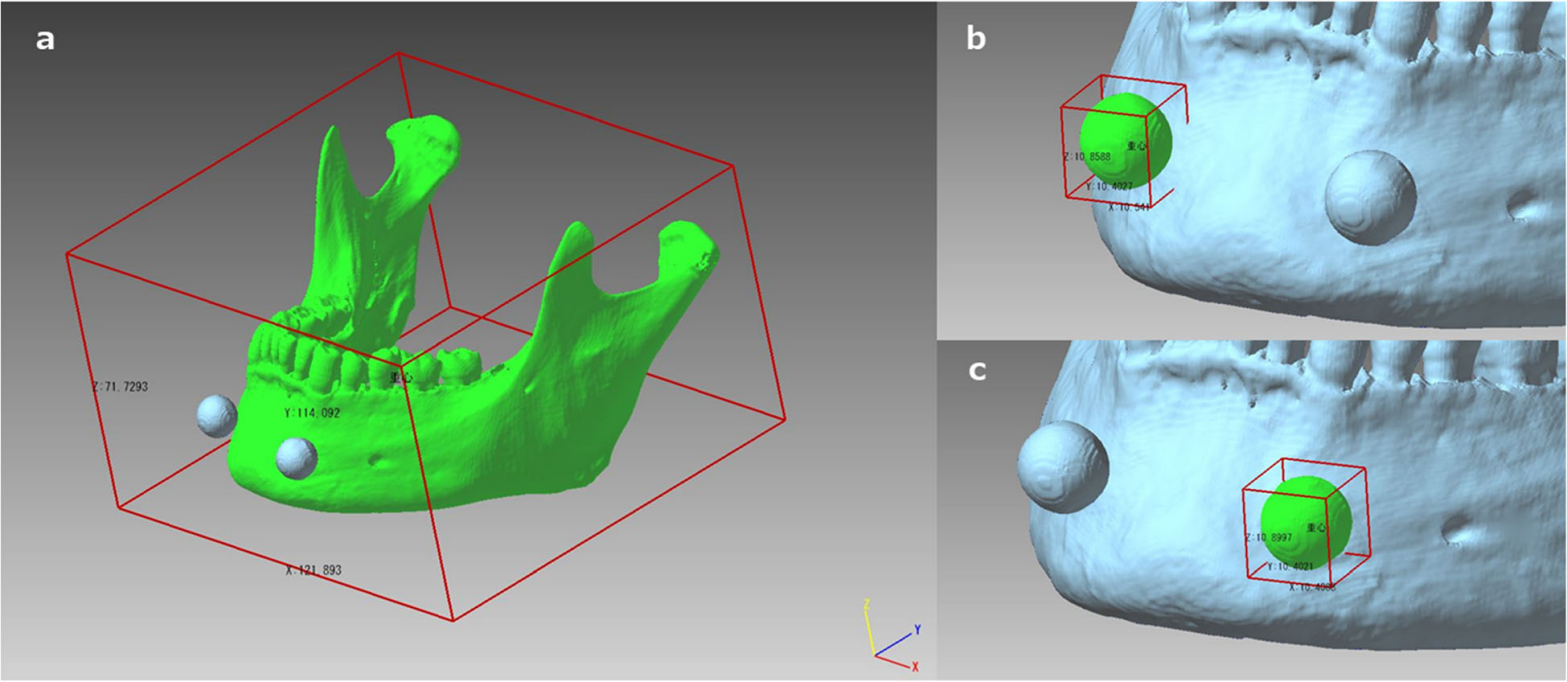
The 3D surface model (virtual 3D model) created from STL data displayed on PMV4. The coordinate system in 3D space, with the length measurement of the STL models in the X-, Y-, and Z-axis directions. Lengths and volumes of the highlighted areas shown in green for the mandible STL model (**a**) and for the ball STL model (**b** and **c**) were measured

Figure 2 shows the coordinate system in 3D space and the measurement of the length of the STL models in the X-, Y-, and Z-axis directions using the polygon editing software POLYGONALmeister Ver. 4 (PMV4, UEL Corp., Tokyo, Japan) [12]. The coordinate system used in this study was based on the DICOM standard; that is, the positive X-axis points toward the phantom’s left side, the positive Y-axis points toward the phantom’s posterior and the positive Z-axis points from the inferior direction to the superior direction.

#### Superimposition and shape error evaluation

To determine the shape error (shape differences between two models that are signed differences), CAD comparison and inspection software SpGauge 2014.1 (SpG, Aronicos Co., Ltd., Shizuoka, Japan) was used for performing the superimposition and measurement. For the superimposition, one of two STL models was moved using the best-fit surface-based registration algorithm of SpG, with the operation repeated until the movement amount with the other STL model approached as close to 0.00 mm as possible. The mean, maximum, and minimum shape errors were recorded, with expansion indicated as positive and contraction indicated as negative. In the color mapping, positive errors were displayed in warm colors, and negative errors were displayed in cool colors.

### Statistical analysis

The Kruskal-Wallis test was performed using the mean absolute deviation of the file size of the data and the number of triangles of the ball STL model and the mandible STL model created from each software package. To determine the tendency of the morphological change when segmenting the STL model from the DICOM images of large and small structures (in this study, large; mandible, small; ball), the correlation between the mandible STL model and the ball STL model was determined using the Spearman’s rank correlation coefficient applied to the difference between lengths in each of the X-, Y-, and Z-axis directions and the differences in volume. Comparisons between the ball STL models were performed by one-way ANOVA followed by Tukey’s multiple comparison test. After superimposition, the shape error of the mandible STL models was evaluated using the Kruskal-Wallis test, and multiple comparisons were performed via the Steel-Dwass test. Statistical analysis was performed using open-source statistical analysis software R Ver.3.6.1 [13], with a statistical significance level set at 5%.

## Results

The data (file) size and the number of triangles were different for each software, and the maximum data (file) size was 71.0 MB, the number of triangles of the mandible STL model was approximately 1.25 million (IN3). The minimum data (file) size was 22.9 MB, the number of triangles of the mandible STL model was approximately 450,000 (MCS) (Table 2). There were no statistically significant differences between the nine software packages for data (file) size and number of triangles.

**Table 2:** Data size and number of triangles for the STL model created by each software package. ^a^Created in binary format

Software package	File size (Megabytes)^a^	Number of triangles	
		Ball STL model^b^	Mandible STL model
3DS	56.3 MB	7468	1,087,868
3DV	55.7 MB	7444	1,086,540
IMJ	55.5 MB	7412	1,074,036
IN3	71.0 MB	7068	1,247,962
MCS	22.9 MB	3212	448,878
MIT	56.1 MB	7468	1,087,612
OSX	55.9 MB	7450	1,081,660
S3D	56.3 MB	7472	1,089,572
VE3	48.3 MB	7380	953,042

^a^Constructed in binary STL format

^b^Mean value of left and right ball STL model measurements

**Fig. 3:**
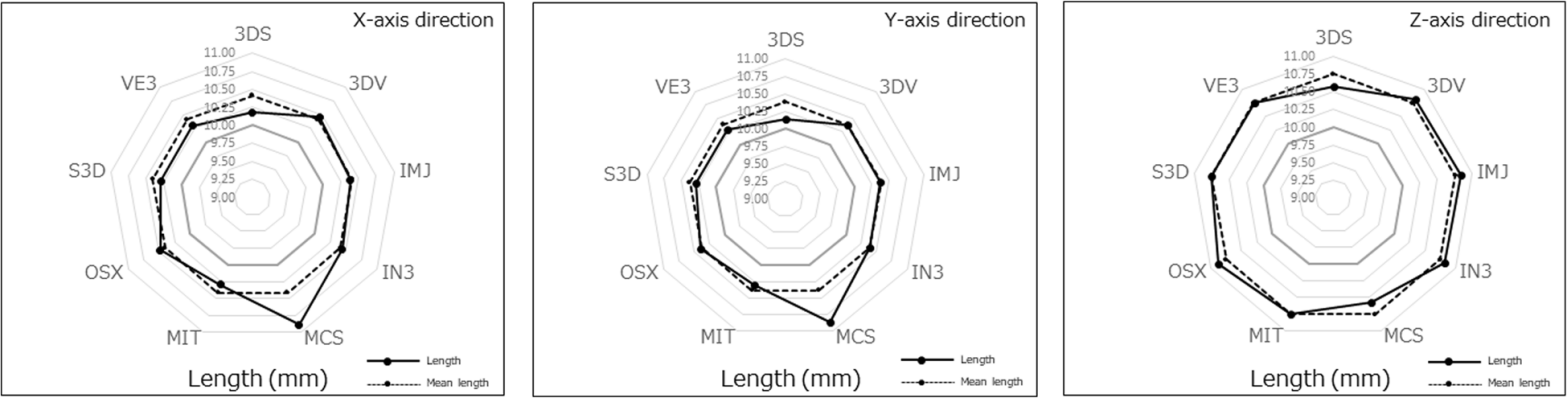
Length measurements of the ball STL model. The solid line indicates the measured value of the length of each ball STL model in the X-, Y-, and Z-axis directions, and the dotted line indicates the mean value of the lengths of all models across all software packages

**Fig. 4:**
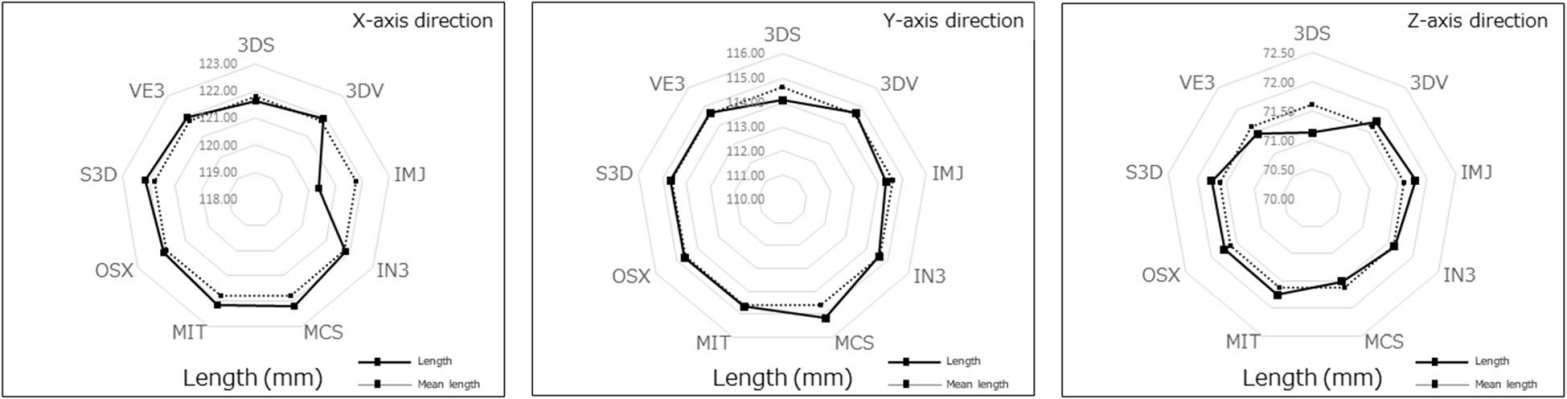
Length measurements of the mandible STL model. The solid line indicates the measured value of the length of each mandible STL model in the X-, Y-, and Z-axis directions, and the dotted line indicates the mean value of all lengths across all software packages

**Fig. 5:**
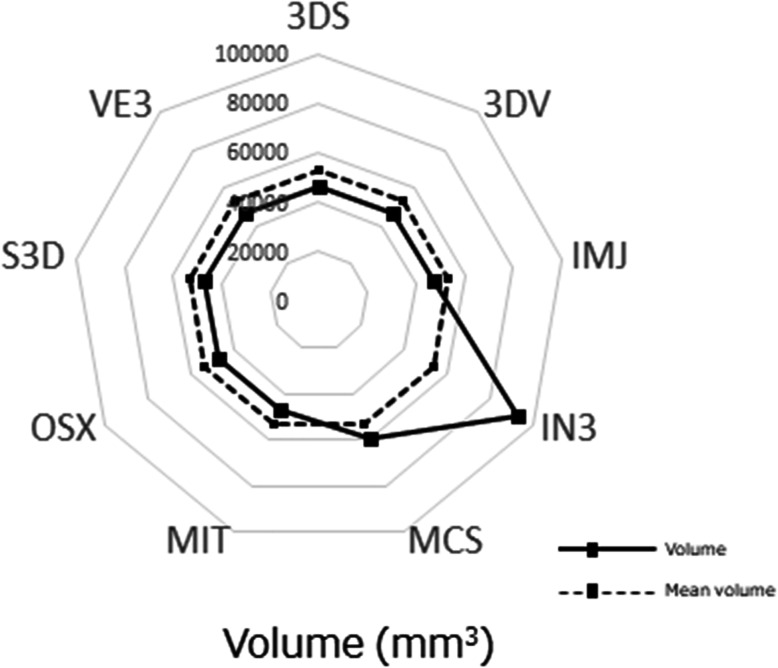
Volume measurements of the ball STL models. The solid lines indicate the measured value of the volume of each STL model, and the dotted lines indicate the mean volume across all software packages

For the ball STL model, the lengths in the X-, Y-, and Z-axis directions exceeded 10 mm, with the length of the Z-axis direction longer than that of the X-, and Y-axis directions, with significant differences between the lengths of the ball STL model across software packages (Fig. 3). One software package (MCS) showed larger length values for the X- and Y-axis directions than the other eight software packages (Fig. 4). A negligible to low correlation was observed between the ball STL model and the mandible STL model for the lengths of the X-, Y-, and Z-axis directions. With regard to volume, a high correlation was found between the ball STL model and the mandible STL model (Table 3). One software package (IN3) showed a larger volume value than the other eight packages (Fig. 5). Evaluation after superimposition of the STL models found slight variations in each software package, with a mean shape error of 0.11 mm, a maximum shape error of + 1.69 mm, a minimum shape error of − 1.55 mm, a median shape error of 0.08 mm and a 95% confidence interval of 0.08 to 0.135. No significant differences were found for the shape error across software packages (standard deviation 0.08, *p-*value 0.393).

**Table 3:**
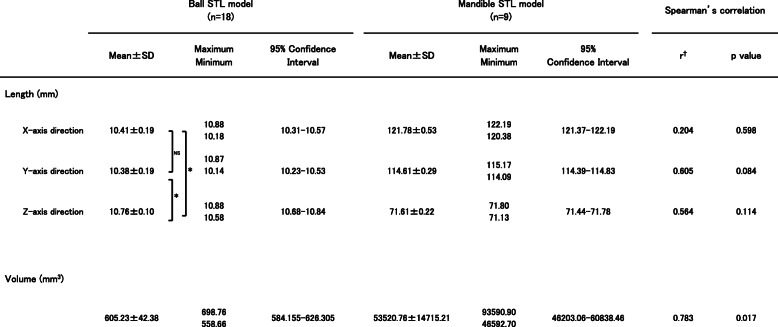
Summary of the length and volume of each STL model. Spearman’s rank correlation coefficient was obtained for each parameter of the ball and the mandible STL models

*p < 0.05.

^†^Correlation coefficient (*r* = 0.00–0.30: negligible correlation, *r* = 0.30–0.50: low correlation, *r* = 0.50–0.70: moderate correlation, *r* = 0.70–0.90: high correlation, *r* = 0.90–1.00: very high correlation)

The number of triangles in the mandible STL model was reduced to 200,000, and their morphological changes were evaluated. The mean shape error of that STL model relative to the models with the largest number of triangles and the mean numbers of triangles was almost 0 mm.

## Discussion

We divided our workflow into three steps, each of which requires a different file format. Step 1 involves acquiring a 3D volume image of the patient as a DICOM image file. Step 2 entails segmenting the anatomical structure from surrounding structures and exporting it to the virtual 3D model in STL file format. The segmentation of osseous structures and soft tissue is relatively easy. However, in many cases, it is difficult to create an STL model for two reasons. One reason is that thin osseous structures (e.g., bone surrounding the nasal cavity, orbital floor) and narrow tissue gaps (e.g., upper and lower joint cavity between the temporal bone and the mandible) are not clearly reproduced in the STL model. Second, many artifacts (e.g., metal artifacts and/or beam hardening from dental prostheses) reduce the readability of the images and prevent segmentation. Step 3 concerns the 3D printing of the physical 3D model, which requires the use of “G-code” generation software to produce G-code as the 3D printable data [14]. Each step of the entire process, namely, the segmentation of DICOM images, the processing of STL data, the generation of G-code data, and the performance of the 3D printer itself, affects the accuracy of the final 3D model. Creating STL data is the most important operation in fabricating the 3D model.

### Characteristics of DICOM segmentation and STL creation software

**Fig. 6:**
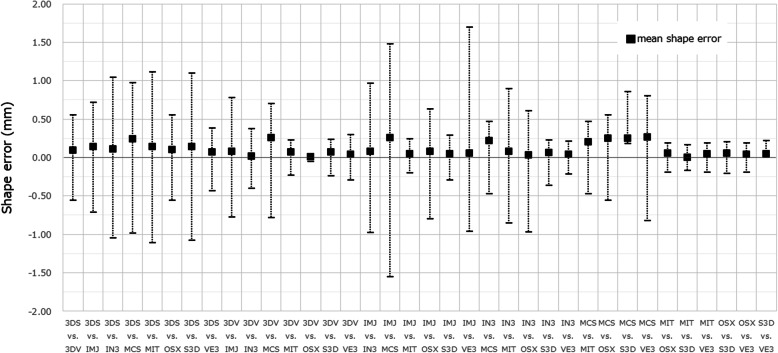
Shape error (signed differences) measurement after superimposing pairs of STL models using SpG. The black square indicates the mean value, the upper limit indicates the maximum value, and the lower limit indicates the minimum value. Multiple comparisons of the shape error of each mandible STL model were performed, and no significant difference was found

**Fig. 7:**
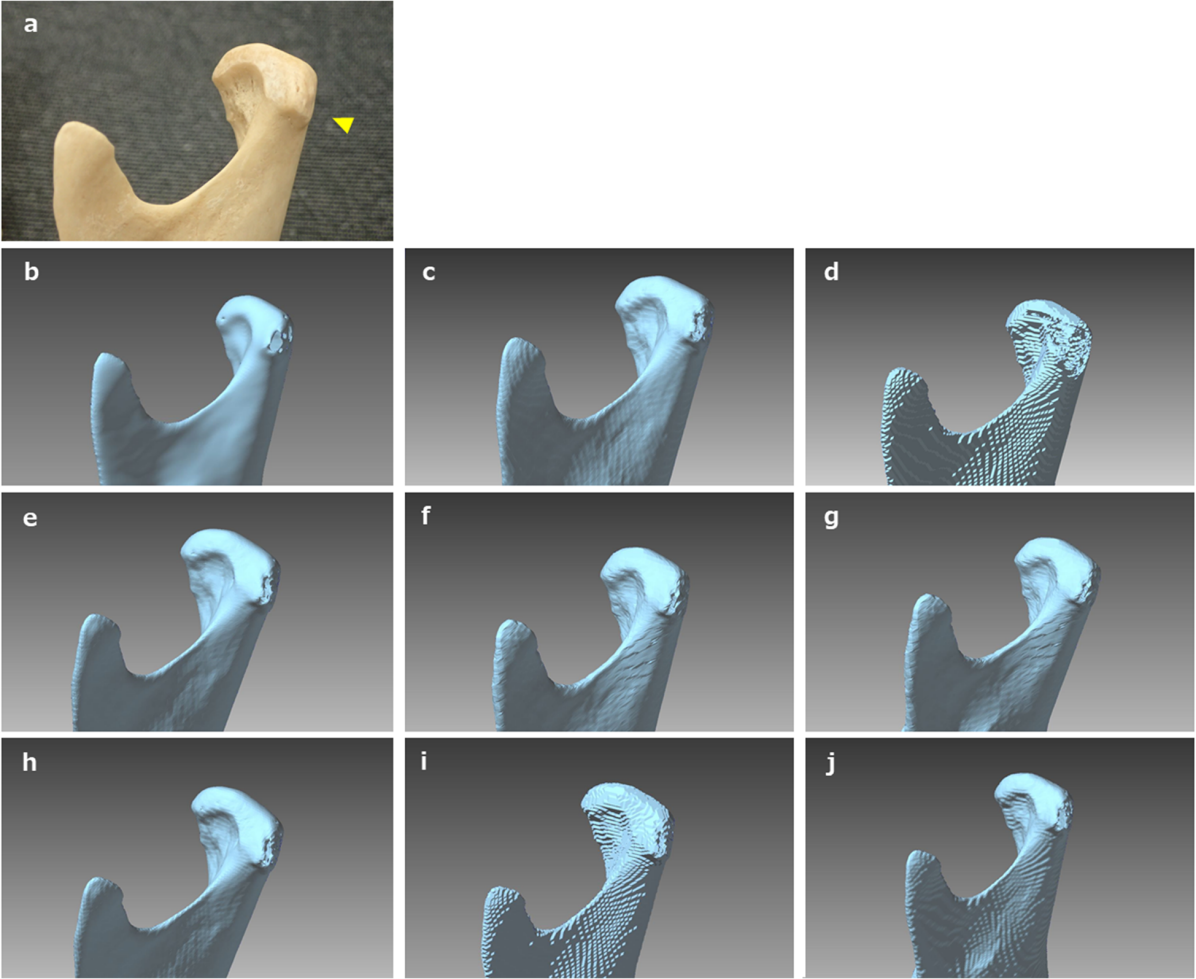
Closer view of the dry human mandibular condyle (**a**), the STL model created from DICOM images using 3DS (**b**), 3DV (**c**), IMJ (**d**), IN3 (**e**), MCS (**f**), MIT (**g**), OSX (**h**), S3D (**i**) and VE3 (**j**). Threshold settings for binarization were the same for all software packages; however, the created surface was slightly different for each model, with differences most notable in thin areas of the cortical bone (arrowhead)

Appearances of the created STL models differed across software packages. Most notably, the cortical bone of the top and/or lateral pole of the mandibular condyle was thin, so the reproducibility of this part was different across all software packages. When “faithfully” fabricating according to this STL model, the steps would appear as holes (defects). Moreover, in some software packages, the surface of each STL model was rough (Figs. 6, 7). Although the ball STL model was created by MDCT scanning of a 10-mm-diameter bearing ball, all software packages rendered it expanded in all directions. The average ball length in all directions was 10.52 mm, but the length in the Z-axis direction was slightly longer than that in the X- and Y-axis directions. This variation is likely because of the differences in the voxel size of the DICOM images (X-, Y-, Z-axis direction lengths were 0.468, 0.468, and 0.500 mm, respectively) and may also have been affected by the partial volume effect that occurred on the border between the ball surface and air. The diameter of the ball in the STL model was calculated from the mean value of the volume (605.23 ± 42.38 mm^3^) to be 10.49 mm. The shape error for this entity was equivalent to the size of one voxel and was reproduced by each software package.

**Fig. 8:**
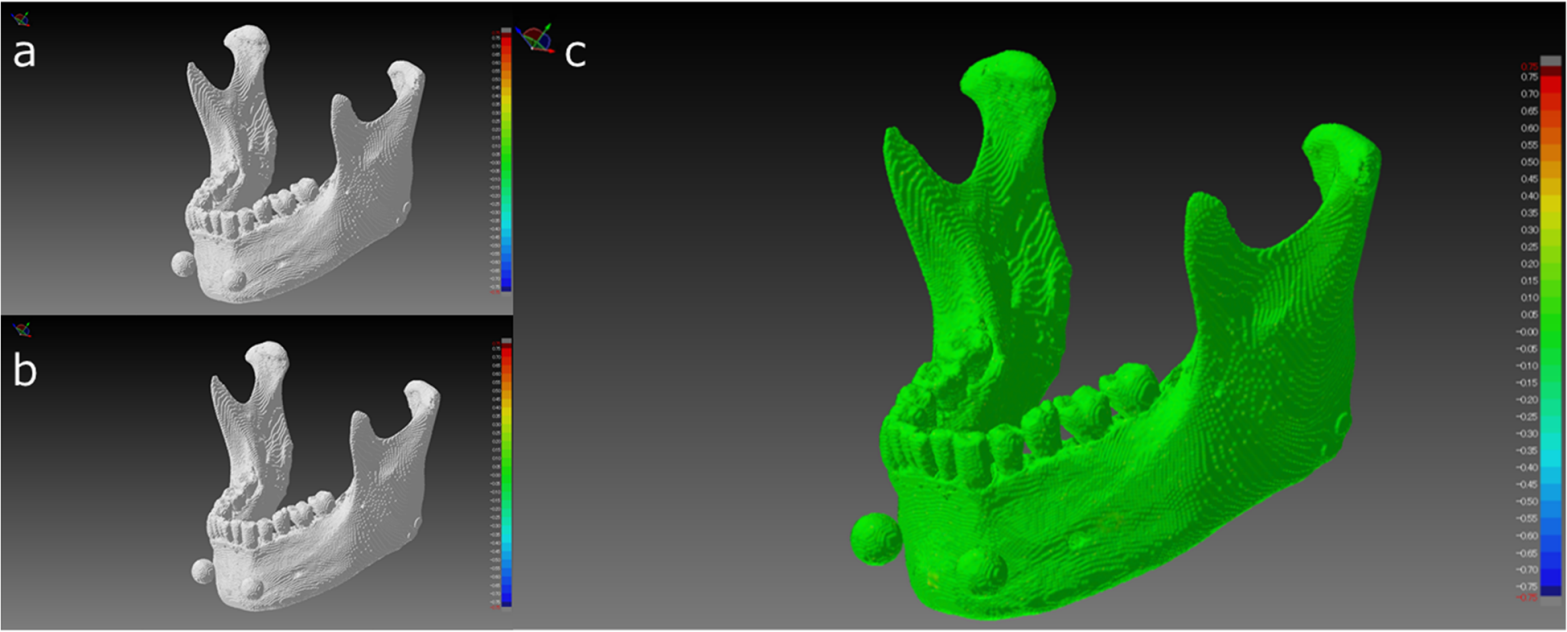
Comparison of STL models between S3D (**a**) and MIT (**b**), where the shape error between the two STL models was the minimum value. Visualization of the shape error (signed differences) after superimposition is shown on the right (**c**). Almost all of the STL model was green. The mean error between the two STL models was 0.00 mm (maximum + 0.16 mm, minimum − 0.17 mm)

**Fig. 9:**
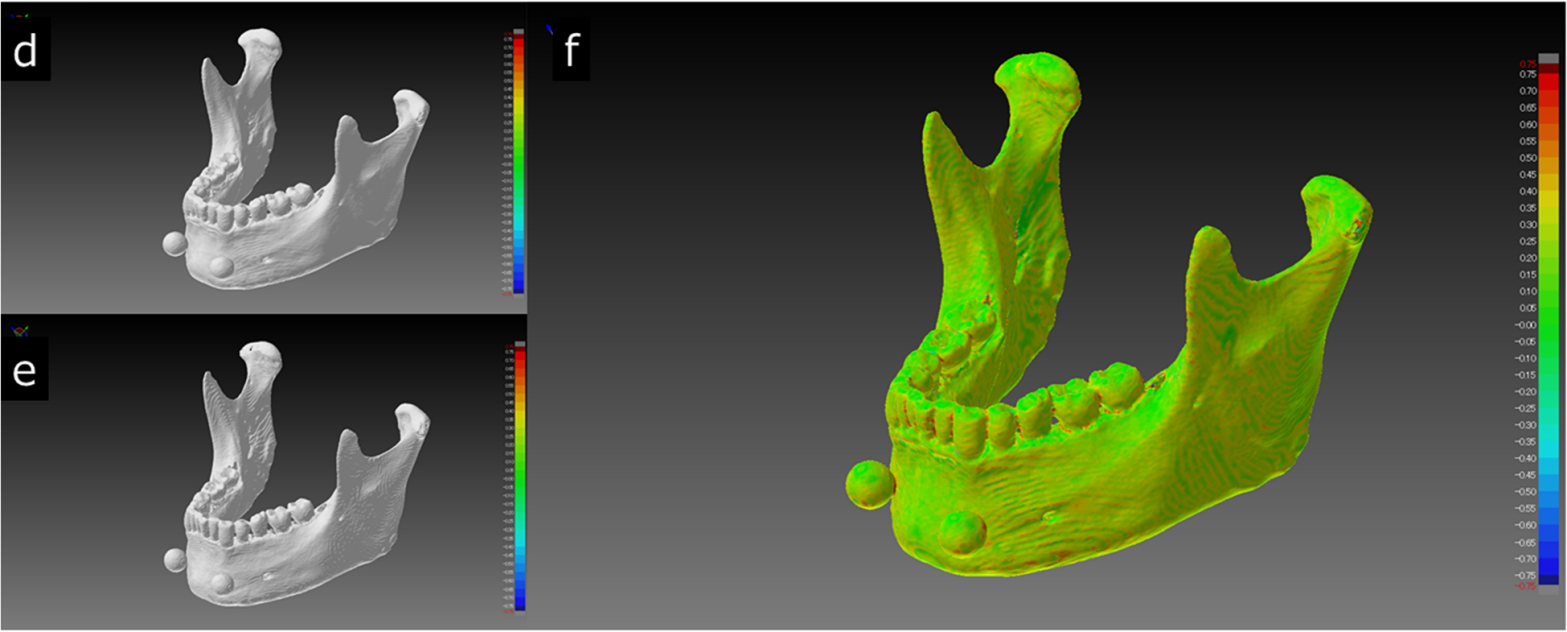
Comparison of the STL model of MCS (**d**) and VE3 (**e**), which showed the largest shape error between any two STL models. Visualization of the shape error (signed differences) after superimposition is shown on the right (**f**). The whole mandible is depicted as green to yellow (shape error range of approximately 0.0 mm - 0.5 mm), with occasional orange to red parts. The mean shape error was 0.27 mm (maximum + 0.80 mm, minimum − 0.81 mm)

It is difficult to quantitatively assess the STL segmentation performance of each software package independently. To solve this problem, we superimposed pairs of STL models (created with different software packages) on each other; the difference between each pair was visualized and measured as a shape error. Although the differences between shapes of the created STL models were visible on the shape error image, no significant differences were found across all mandible STL models. Figure 8 shows images captured by the superimposition and visualization of S3D and MIT, which had the minimum shape error. Figure 9 shows images of MCS and VE3 with a maximum shape error. The reason the shape errors could be seen by the software packages, although only slightly, was that the binarization algorithms differ across the software packages. Binarization means creating an isosurface. The isosurface refers to the boundary surface of the target area formed by setting an appropriate threshold and is generally approximated by a polyhedron as a patch model consisting of a set of fine triangles. The method of creating isosurfaces from volume data is a useful tool and has been used in a wide range of fields, such as the 3D visualization of CT data and modeling of arbitrary shapes by an implicit function expression. A number of methods have been proposed [15–17].

The shape error appeared because of differences in image processing near the threshold values, such as the thin cortical bone or strongly curved surface. The color maps of Figs. 8 and 9 are colored as green to yellow areas, with mean distances of approximately 0.30 mm. This distance is smaller than one voxel size. Regarding the roughness of the surface of the STL model, it was thought that the influence of the unevenness was small. Therefore, it was considered that the shape error was not affected. It is difficult to judge the pass/fail of an error that differs depending on the software package obtained in this study because there is no correct answer. Considering the spatial resolution of MDCT, it can be assumed that this kind of error is acceptable in fabricating 3D models for clinical use in oral and maxillofacial surgery [18–20]. A more thorough analysis of the shape error by region of the mandible could be considered to allow quantification of some of the qualitative findings presented.

### Reducing STL data size

**Fig. 10:**
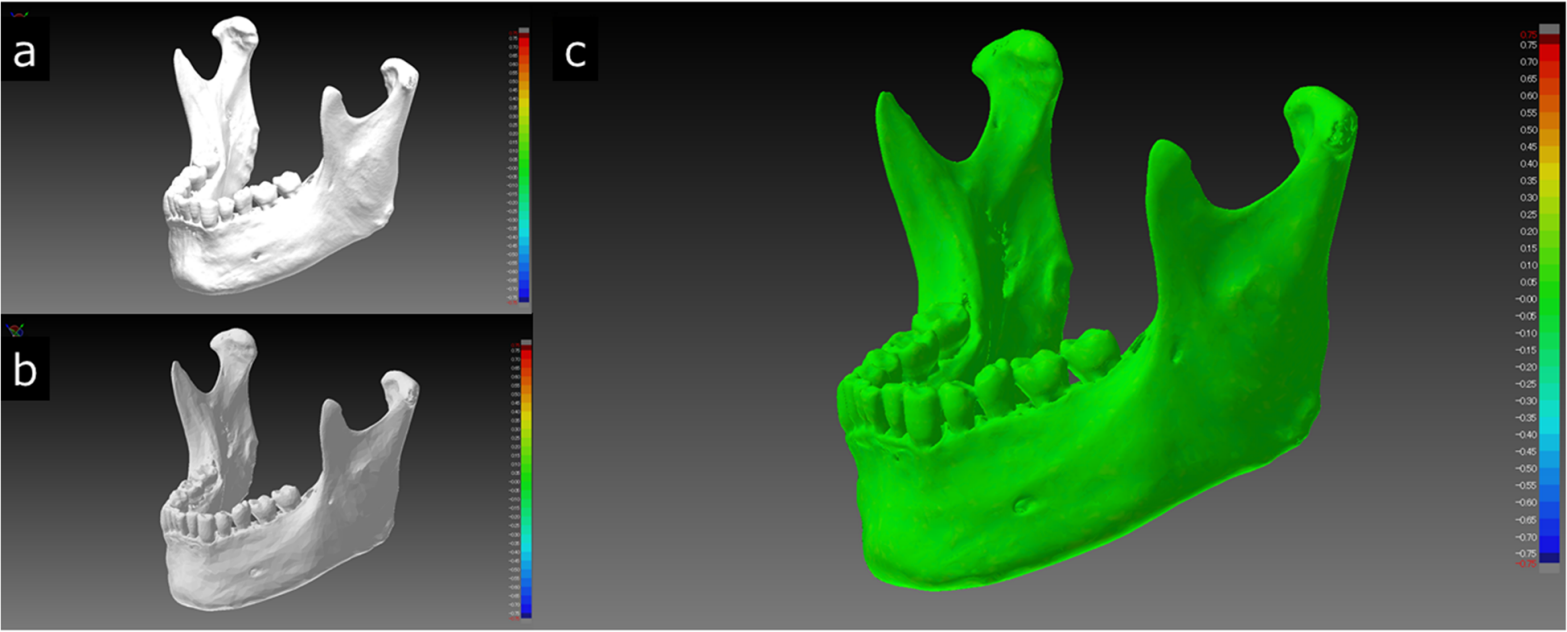
Visualization of the STL model created with IN3, which had the largest volume and number of triangles, the STL model with the reduced number of triangles, and the shape error (signed distances) after superposition. When the original number of 1,247,962 (**a**) triangles were reduced to 200,000 (**b**), the surface of the STL model appeared to be slightly rough. In the color map, the entire area was green (**c**). The mean shape error was 0.02 mm

STL data represent a 3D shape as a collection of small triangles. The number of triangles depends on the size, shape and internal structure of the object. More complex features and higher resolution lead to an increase in the number of triangles in the segmented STL data. Processing a large number of triangles draws heavily on the processing power of a PC; the calculation is time-consuming and can affect subsequent operations. A reduction in the number of triangles directly leads to a reduction in the data size. However, a reduction in the number of triangles may also cause a morphological change [21]. Therefore, the mandible STL model was superimposed before and after the reduction in the number of triangles to evaluate the dimensional change, and the shape error was observed. To reduce the number of triangles to 200,000, i.e., the number of triangles recommended in the report [22], the “simplify data by specifying the number of triangles” function of PMV4 was used [23]. Figure 10 shows the before and after reduction in the number of triangles and the color map after the superimposition of the STL model with the largest volume and number of triangles (IN3; 1.24 million). As a result, although the surface of the STL model with the reduced number of triangles (200,000) was somewhat rough when displayed on the monitor, the mean shape error of that STL model relative to the models with the largest and the mean numbers of triangles was almost 0 mm. It was clarified that data reduction in the mandible STL model of any software package could reduce the data size and did not affect the morphological change. Considering that the minimum laminating pitch of the FDM desktop 3D printer that we use is 0.05 mm (https://www.mutoh.co.jp/3d/doc/product_lineup.pdf), this supports the inference that the recommended number of triangles was both necessary and sufficient for 3D printing.

### Limitations and prospects

There is the opinion that the use of a surface scanner can provide a precise morphological evaluation. A surface scanner was not used in this study. This is because we do not have a scanner, nor do we have the skill to build it. Therefore, we use only the DICOM image data acquired by MDCT scanning to evaluate the accuracy of each software package.

In the evaluation of the data size, the number of triangles, and the morphology of the created STL models, there was a problem that there was no gold standard value. Therefore, we solved this issue by performing multiple comparisons of all STL models. In this study, since only a dry human mandible was used, the segmentation operation with surrounding anatomical structures on a PC, such as soft tissue, was not performed. When performing the 3D printing of a patient’s DICOM data, the segmentation of soft tissues and osseous structures is required. We have no manual measurement (e.g., measurement with a caliper) so it is expected that the measurement results will differ depending on the observer. In addition, optical three-dimensional measurements that require verifying the accuracy of the measurement device itself in advance were not performed.

Shape errors are inevitable because of the spatial resolution limits of MDCT. However, when using 3D models in fields that require more detailed operations, such as microscopic surgery, other modality options should be considered, such as the use of limited cone-beam CT, which is expected to produce a better high-definition STL model. In this study, an MDCT scanner was used to segment DICOM images to STL data under the condition of a fixed voxel value binarization threshold. In addition to differences between patients, physics-based factors such as the irradiation dose and other differences in the MDCT models and scanning parameters may also affect the difficulty of creating STL models [24, 25]. Although no segmentation in the true sense was performed in this study, in the clinical use of 3D printing technology, setting a threshold for 3D printing requires medical knowledge, especially tomographic image anatomy, as well as the knowledge of the modalities of the imaging principles. It seems necessary to understand the features of the software package for STL segments as well.

This study does not aim to rank software packages. There are some differences between DICOM segmentation and STL creation depending on the software, so it is desirable to understand and use these characteristics.

## Conclusions

We evaluated nine commercial/open-source software packages that create DICOM images to STL data. Our evaluation included superimposing STL models created by different software packages over each other to visualize and measure the shape error. Although slight differences were found, the differences were within the slice thickness of the MDCT. In conclusion, when using segmentation software, it is essential to understand the features and characteristics of the software package to align its use with the intended purpose. In creating/designing data for 3D printing of fine and/or thin structures, it is important to pay close attention to setting the threshold for the ROI and for binarizing DICOM images.

## Data Availability

Readers interested in the data should contact the authors.

## Abbreviations

3D: Three (3) dimensional
3DS: 3D Slicer (software package)
3DV: 3DView (software package)
CAD: Computer-aided design
CT: Computed tomography
DICOM: Digital Imaging and Communications in Medicine (file format)
FDM: Fused deposition modeling
IMJ: ImageJ (software package)
IN3: InVesalius 3 (software package)
MCS: Mimics (software package)
MDCT: Multidetector row computed tomography
MIT: The Medical Imaging Interaction Toolkit (software package)
OSX: OsiriX Lite (software package)
PC: Personal computer
ROI: Region of interest
S3D: Seg3D (software package)
STL: Stereolithography (file format)
VE3: Volume Extractor 3.0 (software package)
